# Spontaneous and Engineered Compensatory HSV Mutants that Counteract the Host Antiviral PKR Response

**DOI:** 10.3390/v1030510

**Published:** 2009-10-22

**Authors:** Amish C. Shah, Jacqueline N. Parker, Masako Shimamura, Kevin A. Cassady

**Affiliations:** 1 University of Pennsylvania, Department of Pediatrics, Children’s Hospital of Philadelphia, 34^th^ Street and Civic Center Boulevard, Philadelphia, PA 19104-4399, USA; E-Mail: amishamish@gmail.com (A.C.S.); 2 University of Alabama at Birmingham, Department of Pediatrics, Division of Infectious Disease, 1600 6th Avenue South, CHB 118C, Birmingham, AL 35222, USA; E-Mails: jnparker@uab.edu (J.N.P.); mshimamura@peds.uab.edu (M.S.)

**Keywords:** PKR, oncolytic HSV, Δγ_1_34.5

## Abstract

A virulent recombinant HSV lacking the diploid γ_1_34.5 gene (Δγ_1_34.5) have been investigated over the last two decades both for anti-tumor therapy and as vaccine vectors. The first generation vectors, while safe, are incapable of sustained replication in the majority of treated patients. An interferon inducible host antiviral kinase, protein kinase R (PKR), limits late viral protein synthesis and replication of Δγ_1_34.5 viruses. This review describes the development of new Δγ_1_34.5 vectors, through serial passage selection and direct viral genome engineering, which demonstrate selective PKR evasion in targeted cells and improved viral replication without restoring neurovirulence.

## 1. Introduction

Serial passage is a time-honored viral laboratory method that has been used to select progeny with attenuating mutations (e.g. OKA vaccine strain from varicella zoster virus). In recent years, serial passaging has been used as a method to identify secondary mutations in attenuated HSV oncolytic vectors that mediate improved viral replication and anti-tumor activity. Additional uses for the serial passaging techniqueinclude: *i*) the testing of genetic stability of biologic therapeutics and potential pathogenic mutations; *ii*) identification of cryptic gene functions that may have served evolutionarily distant functions for the virus; and *iii*) to identify antiviral escape mutations, that can then provide leads to improved drug design.

This review focuses on the interferon-induced, innate antiviral response triggered during viral transcription that limits protein synthesis initiation in the infected cell. In addition to discussing the protein product encoded by the HSV γ _1_34.5 gene that counters this host antiviral response, the review also describes how mutant viruses lacking the γ_1_34.5 gene (Δγ _1_34.5) have been studied as vaccine and anti-tumor therapies because of their safety in humans. While these vectors are safe, their limited late viral protein synthesis and diminished replication limit their efficacy. Serial passage of Δγ _1_34.5 recombinants has been used to identify whether compensatory mutations improve viral replication or the anti-tumor properties of the Δγ_1_34.5 mutants. Current efforts to improve these vectors’ replication are now focused upon engineering a new generation of HSV recombinants based on the earlier serial passage experiments.

## 2. PKR Innate Antiviral Response

**Figure 1.**
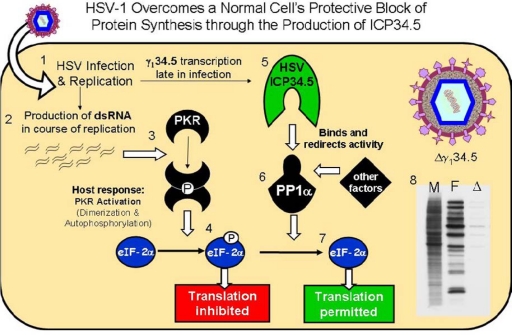
Summary of PKR mediated protein shutoff and HSV ICP34.5 defense. (1) HSV entry, gene expression, 2) complementary transcripts anneal and form stable dsRNA 3) PKR binds dsRNA resulting in activation and autophosphorylation, 4) activated PKR selectively phosphorylates eIF-2α, inhibiting protein translation, 5) HSV produces ICP34.5 which 6) forms a multiprotein complex with the host phosphatase PP1α 7) and dephosphorylates eIF2α. 8) An example of pulse labeling study showing abundant radioactive methionine incorporation in uninfected (M) and wild-type HSV-1-infected (F) proteins at 12hpi but decreased incorporation in ICP34.5(-) infected cells (Δ).

Eukaryotic cells contain innate defenses that target viral infection [1]. A principal component of this defense is protein kinase R (PKR), which limits viral gene expression and replication in human cells (summarized in Figure 1) [2].

Low levels of this evolutionarily conserved, interferon-inducible kinase are present in unstressed cells. However, its production is induced by type I interferons or double-stranded RNA (dsRNA) produced during viral infection [1]. Upon binding dsRNA, the kinase activates and phosphorylates itself as well as cellular proteins involved in the antiviral response. The best characterized of the substrates is the α subunit of eukaryotic translation initiation factor 2 (eIF-2α) [3,4]. Phosphorylation of eIF-2α prevents recycling of a critical translation initiation factor, thus limiting viral and cellular protein synthesis in the infected cell [2].

In addition to its antiviral functions, the PKR-mediated host protein shutoff response is also involved in cellular homeostasis. Consequently, second messenger signaling pathways and PKR modulate one another’s activity in the cell. These pathways can block PKR activation during periods of cellular stress or when the cell is actively replicating. For example, upregulation of mitogen-activated protein kinase (MAPK) activity in the cell (mediated by a component of this pathway, MEK) blocks PKR activation during growth factor stimulation [5,6]. Likewise, PKR has also been shown to act as a signal integration point between signaling pathways [7]. While regulation of protein synthesis initiation is the best characterized of the PKR antiviral functions, the kinase also modulates other cellular functions, including: bulk protein degradation in the cell (also called autophagy), RNA transcription, and signal transduction in the cell [5,8–10].

## 3. The HSV-1 γ_1_34.5 Gene

The HSV-1 γ_1_34.5 gene encodes a multifunctional protein called ICP34.5 [11,12]. One function, encoded within the 3′ gene domain, blocks host PKR-mediated protein shutoff during infection thus allowing continued late viral protein synthesis in infected cells [13]. During infection, wild-type HSV-1 produces complementary mRNA transcripts that anneal, forming stable dsRNA, which triggers the dimerization and activation of dsRNA-activated host PKR. The ICP34.5 overcomes this PKR-mediated host protein shutoff by binding and recruiting a host phosphatase that specifically dephosphorylates eIF-2α, allowing continued viral protein synthesis (hereafter referred to as the HSV wild-type protein synthesis phenotype) in the infected cell (summarized in Figure 1) [14,15].

Recombinant viruses that lack the γ_1_34.5 gene, (Δγ_1_34.5 HSVs) are incapable of maintaining eIF-2α in an unphosphorylated form and therefore are unable to maintain protein synthesis in the infected cell [16]. Cessation of protein synthesis occurs at the onset of viral DNA synthesis late in infection, essentially eliminating bulk synthesis of viral structural proteins necessary for viral capsid formation [16]. In addition to structural proteins, viral encapsidation, envelopment and maturation are necessary for efficient viral replication. There are examples of Δγ_1_34.5 mutants that are capable of late viral protein synthesis but that cannot negotiate the other steps necessary for viral replication [17]. Evidence suggests that in addition to protein translation, other cellular processes are disrupted in Δγ_1_34.5 infected cells that are critical for viral egress. Consequently, Δγ_1_34.5 HSV replicate inefficiently and produce fewer progeny virions in cells with intact PKR pathways [18]. While the γ_1_34.5 gene product does not interrupt IFN signaling in the infected cell as has been described with the HSV U_L_13 and U_L_39 gene products, ICP34.5 is critical for HSV evasion of the IFN inducible gene product, PKR. Consequently, Δγ_1_34.5 viruses are highly sensitive to Type I IFN and replicate poorly *in vivo* or in IFN treated cells [19,20]. Restoration of the protein synthesis function allows Δγ_1_34.5 HSV to replicate efficiently in the presence of the type 1 IFN (α or β interferon) response [17].

The γ_1_34.5 gene also encodes a second function, neurovirulence, enabling efficient viral replication in post-mitotic neuronal cells [12–14,21]. The neurovirulence and protein synthesis functions encoded by the γ_1_34.5 gene are discrete and separable. Late viral protein synthesis can be selectively restored without restoring wild-type neurovirulence [17,22,23]. The Δγ_1_34.5 HSV are incapable of efficient replication after direct inoculation in the CNS and do not cause encephalitis [12]. As such, Δγ_1_34.5 HSV vectors have been developed as anti-tumor agents for CNS-based malignancies. Whereas 50–100 PFU of wildtype HSV will lead to encephalitis and death in half of the mice inoculated intracerebrally, more than 1 × 10^7^ PFU are required to produce encephalitis and death with a Δγ_1_34.5 HSV recombinant [12]. Finally, the γ_1_34.5 gene also allows HSV-1 to block autophagy in infected cells [24]. This function contributes to HSV neurovirulence, maps within the 5′ domain of the gene and allows binding and sequestering of beclin-1, a host protein necessary for autophagosome formation [24].

## 4. Δγ_1_34.5 HSV as Oncolytic Vectors

Genetically modified HSV are attractive as replication-competent, oncolytic vectors as well as vaccine vectors for a number of reasons: 1) procedures for constructing novel HSV are well established; 2) genetic modifications (insertions or deletions) do not significantly affect virus replication; 3) considerable experience with the biology of HSV and its behavior in humans and nonhuman primates exists; and 4) modified herpesviruses retain sensitivity to standard antiviral drug therapy as a “built-in” safety feature [25–27]. Deletion of the HSV-1 neurovirulence gene, γ_1_34.5, allows the safe administration of recombinant vectors intracranially (for brain tumor therapy) or systemically (for peripheral tumors or as a vaccine vector).

**Figure 2.**
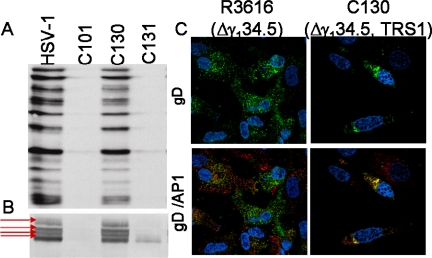
Glycoprotein processing differences between Δγ_1_34.5 and HSVs capable of PKR evasion and late viral protein synthesis. A) Autoradiograph showing radiolabeled viral proteins produced in infected malignant glioma cells. Viruses capable of PKR evasion (HSV-1 and the chimeric HSV, C130 [discussed in detail in section 6.4]) produce abundant ^35^S-Methionine-radiolabeled proteins at 12hpi. In contrast, the Δγ_1_34.5 recombinants (C101) and the Chimeric HSV repair virus (C131) have reduced protein synthesis. B) Immunostaining using Ig anti-glycoprotein D (clone H170: SC-69802 Santa Cruz Biotechnology) shows abundant late gene accumulation in the HSV-1 and chimeric HSV (C130) samples as well as multiple migratory gD forms (arrows) indicative of glycoprotein processing in the infected cells. C) Confocal immunofluorescence imaging shows gD (green) distribution in Δγ_1_34.5 (left upper panel) and C130 (right upper panel) -infected U87-MG cells and colocalization with the Transgolgi network marker AP-1 (Sigma). Co-localization detected (yellow) in the chimeric HSV infected samples (lower right panel) but not in the Δγ_1_34.5 infected cells.

In addition to inhibiting protein synthesis initiation, other viral functions are inhibited as a consequence of this deletional mutation. The Δγ_1_34.5 mutants also demonstrate changes in glycoprotein processing Figure 2). The HSV γ_1_34.5 gene product differs in amino acid sequence between viral strains. Adoptive transfer studies have shown that these ICP34.5 amino acid differences affect glycoprotein processing and plaque phenotype in cell culture [28,29]. In viruses lacking the γ_1_34.5 gene, the differences in glycoprotein processing are even more dramatic. Immunostaining studies show that gD in the Δγ_1_34.5 infected cells exist in a single form after separation using denaturing conditions. In contrast, viruses capable of PKR evasion and late viral protein synthesis exhibit multiple slower migratory forms of the glycoprotein indicative of further glycoprotein processing. In figure 2 the chimeric HSV, C130, which contains the HCMV PKR-evasion gene TRS1, contains multiple forms of glycoprotein D, similar to that observed for wild-type HSV. Confocal immunofluorescence microscopy further demonstrates differences in glycoprotein trafficking in the infected cell. In tumor cells infected with recombinants capable of late viral protein synthesis, gD accumulates within the trans golgi network (TGN) and late endosomal compartment based upon gD and AP-1 staining and colocalization. In contrast, gD expressed in the Δγ_1_34.5 infected cells does not localize with adaptor protein complex 1 (AP-1) in the TGN. There are also fundamental differences in the appearance of the TGN. In the Δγ_1_34.5 infected cells, the TGN appears disrupted throughout the cytoplasm and does not exhibit the more structured perinuclear aggregates seen in cells capable of continued protein synthesis. The Δγ_1_34.5 recombinants also exhibit differences in protein degradation or autophagy within the infected cell [8]. Improved protein production and processing may may enhance the antitumor capabilities of Δγ_1_34.5 mutants either directly (improved antigen expression and replication) or indirectly by improving the expression and processing of foreign gene inserts in recombinants for gene therapy applications.

In addition to protein processing recent studies also show that the γ_1_34.5 gene product also inhibits IFN response in the cell. ICP34.5 targets the ability of the TANK-binding kinase 1 (TBK1) to activate an antiviral response through interferon regulatory factor 3 (IRF3) and cytokine expression. [30] Phosphorylation and transnuclear translocation of IRF3 as well as expression of downstream genes functioning in antiviral responses are thereby inhibited by the γ_1_34.5 protein product. Suppression of the interferon signaling pathway has consistently been shown to be a major mode of the interaction of HSV with the innate immune system, and its interaction with TBK1 appears to be a novel but critical mechanism to ensure successful and productive HSV infection.

## 5. Serial Passage and Escape Mutations

### 5.1. *In vitro* passage

Attenuated Δγ_1_34.5 recombinants have been engineered for use as antitumor agents. While the vectors are safe and replicate in mitotically active cells, they do not exhibit robust replication. This limited replication and spread is one factor thought to limit their efficacy *in vivo*. Studies have described acquisition of mutations that overcome certain limitations of the ICP34.5-negative status of attenuated HSV-1 [31]. Mohr and Gluzman reported selection of the SUP-1 mutant after passage of the Δγ_1_34.5 SPBg5e (derived from the Patton HSV-1 strain) in the nonpermissive SK-N-SH human neuroblastoma cell line *in vitro* [31]. SUP-1 acquired a deletion in the US12 region, resulting in two fundamental changes in the viral phenotype: 1) deletion of U_S_12 results results in greater MHC I expression (as discussed in detail in Section 6), and 2) earlier expression of the US11 gene [31,32]. Later studies using engineered recombinants and recombinant expressed U_S_11 protein in biochemical studies demonstrated that this kinetic shift in U_S_11 expression enabled viral evasion of PKR and late viral protein synthesis [33]. The U_S_11 product, although capable of precluding activated PKR from phosphorylating its target substrate eIF-2α, was much more effective and at lower concentrations at blocking the activation of PKR by dsRNA [32]. Ultimately these escape mutations produced progeny virus capable of continued viral protein translation in infected SK-N-SH cells [33]. Mohr’s mutant SUP-1, while it had a protein synthesis phenotype similar to that of wild-type HSV-1, retained the neurovirulence profile of its Δγ_1_34.5 parent (LD_50_>6 ×10^5^); however, it was engineered in a more neurovirulent strain of HSV-1 which limited maximal LD_50_ testing [34,35].

R3616, derived from the parent HSV-1 (F) strain, was also sequentially passaged in two different cell lines that restricted Δγ_1_34.5 late viral protein synthesis. Serial passage in HeLa cells produced progeny virus with no compensatory mutations [18]. The parent passage 0 virus and the virus recovered after 8 serial infections in the HeLa cell line both had a Δγ_1_34.5 phenotype. It was concluded that because Δγ_1_34.5 recombinants are capable of modest viral replication in HeLa cells that the selection was not sufficiently rigorous to provide compensatory mutant viruses with a selective advantage. Serial passage in SK-N-SH cells produced progeny that, like SUP-1, were capable of blocking the protein kinase R (PKR)-induced shutoff of protein synthesis [18]. Unlike SUP-1, however, these mutations mapped outside of the U_S_10–12 domain, and at least one isolate had acquired a partially restored neurovirulence profile [18].

### 5.2. *In vivo* passage

*In vivo* passage has also been used to develop improved Δγ_1_34.5 anti-tumor activity. M002, a Δγ_1_34.5 recombinant that expresses murine IL-12, was sequentially passaged in flank tumors of the HSV-1 resistant D54-MG cell line. This study was based on the assumption that the virus would, through selective pressure incurred by the tumor microenvironment, acquire mutations allowing for more efficient replication in the resistant tumor cell line [36]. M002 and its parent virus, R3659 were each sequentially passaged in D54-MG tumor cells *in vivo* in flank tumors, and *in vitro* in cell culture [36]. *In vitro* passage in tissue culture selected for mutants similar to the passaged virus described originally by Mohr. The progeny virus expressed U_S_11 earlier in infection, exhibited a wild-type protein synthesis phenotype, and was found in *in vivo* studies to partially restore HSV neurotoxicity (LD_50_ - 5.1×10^5^ – 1.67×10^6^). Interestingly, these secondary mutations did not improve the anti-tumor activity of the Δγ_1_34.5 recombinants. Tumor-bearing animals had similar survival rates irrespective of whether they were administered the parent virus or a serially passaged virus capable of late viral protein synthesis [36].

In contrast, *in vivo* passage in D54 flank tumors selected for second site Δγ_1_34.5 mutants with different compensatory mutations and a different phenotype. *In vivo* passaged viruses demonstrated decreased neurovirulence as compared to the non-passaged parent viruses and resulted in significantly improved survival of tumor-bearing mice in two separate murine experimental brain tumor models: human D54-MG intracranial xenografts in SCID mice and murine Neuro-2a neuroblastoma syngeneic tumors in A/J mice. The *in vivo* passaged virus retained strict γ_2_ kinetic expression of the U_S_11 gene and Δγ_1_34.5 protein shutoff phenotype. However, the recombinants were more effective in their anti-tumor activity [36]. In contrast to specifically engineered constructs, it can be more difficult to identify the particular mutation(s) responsible for the phenotypic changes seen in passaged viruses, and therefore, their interaction with the host environment more elusive to explain. Currently, the compensatory mutation responsible for this change has not been mapped, re-emphasizing the primary limitation of the serial passage process. In cases where a deletional or insertional mutation is responsible, mapping the mutation can easily be achieved through restriction enzyme digestion and differences in DNA fragment migration in agarose gels. In cases where a point mutation or a small deletion mutation occurs, sequencing of the viral genome or using marker transfer of genetic domains must be used to map the mutation. Thus, these studies emphasize the importance of the *in vivo* tumor environment for selecting novel oncolytic HSV strains that mediate improved survival *in vivo*.

## 6. Engineered Mutations

The serial passaging of many viruses has resulted in selection of novel mutations providing unique insights into the complexity of evasion of PKR by HSV vectors. The findings of these studies have helped investigators specifically engineer viruses with the goal of improving their anti-tumor activity and producing more effective vaccine vectors. Additionally, other strategies have been investigated which involve the engineering of novel viruses to enhance the oncolytic activity of Δγ_1_34.5 HSV oncolytic vectors. These are summarized in the following subsections.

### 6.1. R8309

One strategy employed replacing the carboxyl terminus of the γ_1_34.5 gene, responsible for blocking host PKR-mediated protein shutoff, with the murine gene encoding myeloid differentiation gene 116 (MyD116), which is homologous to this region [13]. Both MyD116 and the hamster homologue GADD34, are primary response genes in hematopoiesis and expressed during growth arrest and DNA damage. The virus constructed with this substitution in the γ_1_34.5 gene, R8309, does not induce premature host protein shutoff in infected human cells [13]. Treatment of human gliomas established intracranially in SCID mice with R8309 prolonged survival as compared to controls [22].

### 6.2. GΔ47 – engineering the ΔU_S_12 αU_S_11 mutation

Tumor destruction is expected to be determined in part by the properties of the oncolytic virus used, but is also likely strongly influenced by viral-host interaction and induction of an immune response against the tumor. U_S_12 gene expression from HSV-1 functions to inhibit transporter associated with antigen presentation (TAP) of MHC I. When deleted, MHC Class I expression from infected cells is increased. The GΔ47 virus contains an engineered α47 gene deletion in a G207 vector [37]. Like the second site mutants described above, it places U_S_11 gene expression (normally a γ or late gene) under the control of the immediate-early α47 promoter [33]. The authors suggest the combination of early U_S_11 expression (allowing for improved viral replication) along with increased MHC I presentation (allowing enhanced cytopathic effect) result in greater anti-tumor activity of GΔ47 in various animal tumor models, including metastatic breast cancer to the brain [38], prostate adenocarcinoma [39], and schwannomas [40]. These benefits were achieved without increasing the neurovirulence of the vector [37].

### 6.3. Controlled expression of the γ_1_34.5 gene or tumor targeting of γ_1_34.5 containing HSV

Deletion of the γ_1_34.5 neurovirulence gene allows the safe administration of oncolytic HSV to brain tumors without producing hemorrhagic encephalitis characteristic of wild-type HSV-1 infection. However, since this deletion also diminishes the replicative and cytotoxic abilities of the virus, groups have investigated the targeted expression of the γ_1_34.5 gene to replicating tumors. One method involves placing the γ_1_34.5 gene expression under the control of a tumor specific promoter or enhancer thereby limiting γ_1_34.5 gene expression in complementing tumor cells. One copy of the γ_1_34.5 gene was reintroduced under the control of the cellular B-myb promoter, which responds to E2F regulation [41]. As expected, late viral protein synthesis was maintained after infection with this Myb34.5 virus, which also has a deletion of the ICP6 ribonucleotide reductase gene, and there was greater viral replication. Anti-tumor effect was increased relative to Δγ_1_34.5 HSV-1 in a murine model of liver metastasis [42]. More importantly, the study suggests the potential of regulating γ_1_34.5 gene expression by promoters regulated by the cell-cycle to permit HSV-1 to demonstrate its efficacy within cycling tumor cells yet retaining an advantageous neurovirulence profile. Further development of the targeted expression of the γ_1_34.5 has led to the development of two other conditional expressing HSV recombinants that regulate γ_1_34.5 gene expression using nestin enhancers or musashi promoter (both active in GBM tumors and CNS stem cells) [43,44]. Another method is to limit HSV (γ_1_34.5 +) viral entry to tumor cells. To achieve this, recombinant R5111 was created and replaces the HSV entry molecule gD with a tumor specific fusion entry molecule (gD-IL-13 R α2) [45]. Cell culture studies show that R5111 replicate specifically in tumor [46].

### 6.4. Chimeric HSV

A different method hypothesized that transfer of the human cytomegalovirus (HCMV) PKR evasion genes TRS1 or IRS1 gene to a Δγ_1_34.5 virus (producing viruses C130 and C134, respectively) would improve viral replication and anti-glioma activity. The TRS1 and IRS1 protein products are HCMV’s tools for blocking the dsRNA-dependent PKR response pathway [47,48]. These “chimeric” HSV vectors expressing HCMV genes improved late viral protein synthesis by preventing the shutdown of protein synthesis after viral infection. This attribute proffers the ability to replicate to wild-type levels in human malignant glioma cells *in vitro. In vivo*, C130 and C134 were found to be aneurovirulent with LD_50_ measurements from four to over six logs higher than that of wild-type HSV-1 (LD_50_ = 6.8×10^5^ for C130, >1×10^7^ for C134) [23]. Therefore, despite restoration of late viral protein synthesis and greater replication of progeny virus, expression of TRS1 and IRS1 did not restore wild-type neurovirulence. Indeed, incorporation of TRS1 and IRS1 into the attenuated Δγ_1_34.5 virus produced a more robust vector, permitting more efficient destruction of tumor cells and resulting in enhanced anti-tumor activity. Improved survival was noted in two brain tumor models: a human malignant glioma in severe combined immune deficient (SCID) mice and a syngeneic immunocompetent murine neuroblastoma model [23]. Both chimeric viruses demonstrated enhanced anti-glioma activity compared to their parent Δγ_1_34.5 virus, and performed superiorly at lower doses. A similar greater anti-tumor benefit was seen comparing the viruses against a murine neuroblastoma model [23]. These studies suggest that replication of oncolytic HSV-1 vectors in partially restrictive tumor cells due to anti-viral PKR responses can be significantly improved by encoding PKR-evasion genes from a related herpesvirus.

## 7. Conclusions

Viral protein synthesis is critical for efficient viral replication. As a consequence, HSV has evolved numerous genes involved in maintaining cellular functions necessary for viral replication. Despite the extensive viral control of the cellular environment, intrinsic intracellular defense systems are nevertheless active and can disrupt viral gene expression. Efforts over the last decade have translated some of the principal work involving HSV innate immune evasion toward development of HSV-1 viral vectors for use as both vaccine and anti-tumor therapy. Fundamental scientific research elucidating these viral mechanisms has made these clinical applications possible.
